# The complete mitochondrial genome of *Meretrix lusoria* (Bivalvia: Veneroida: Veneridae) from Kumamoto, Japan

**DOI:** 10.1080/23802359.2020.1778555

**Published:** 2020-06-16

**Authors:** Chia-Hsuan Sung, Sheng-Tai Hsiao, Liang-Jong Wang, Yasuhisa Henmi, Chang-Wen Huang

**Affiliations:** a Planning and Information Division, Fisheries Research Institute, Keelung, Taiwan; b Department of Aquaculture, National Taiwan Ocean University, Keelung, Taiwan; c Marine Fisheries Division, Fisheries Research Institute, Keelung, Taiwan; d Forest Protection Division, Taiwan Forestry Research Institute, Taipei, Taiwan; e Center for Marine Environment Studies, Kumamoto University, Kumamoto, Japan

**Keywords:** Mitochondria, mitogenome, Asia hard clam, *Meretrix lusoria*

## Abstract

We sequenced and assembled the complete mitochondrial genome sequence of the *Meretrix lusoria*, from Kumamoto, Japan. The length of mitogenome is 20,180 bp, including 13 protein-coding genes, two ribosomal RNA genes, and 22 transfer RNA genes. The nucleotide composition of the mitogenome was 25.73% for A, 42.41% for T, 9.35% for C, and 22.49% for G. The AT and GC skewness of mitogenome sequence are −0.245 and 0.412, showing the T-skew and G-skew. The reconstructed phylogenetic relationships of 25 Bivalvia species based on 12 protein-coding genes were highly supported and the clade of all *Meretrix* clams included had a support value of 99%. Our results shall provide a better understanding in the evolutionary histories of the Veneroida and relative species.

The *Meretrix* clams are widely distributed along the coast of East Asia to Southeast Asia. Three *Meretrix* species, *Meretrix lusoria*, *M. lamarckii*, and *Meretrix* sp. are distributed in Japan. The *M. lusoria* and *M. lamarckii* can be found in the shell mounds of the Jomon period about 8000 years ago (Sakatsume 1959). According to the survey on marine fishery production, *M. lusoria* was an important commercial bivalve in Japan, particularly in Tokyo Bay, Ise Bay, and the Ariake Sea (Henmi et al. 2014). Although the abundances of the clams have decreased greatly in many regions, the Shirakawa river and Midorikawa river are still the important habitats for the natural population (Nakamura 2013; Hashiguchi et al. 2014). However, it is difficult to identify the hard calms only depending on the morphological characteristics. The DNA molecular information is useful and easy to identify them correctly and quickly, especially the sequence of mitochondrial DNA.

In this study, the Asia hard clam was collected from Shirakawa river (32°46′36″N; 130°36′42″E) in Kumamoto, Japan and stored in Fisheries Research Institute in Keelung, Taiwan with accession number FIRM10017. The total of 4.3 Gb next-generation sequencing paired-end reads was used to assemble the complete mitogenome sequence of the *M. lusoria*. The CLC Genomics Workbench (QIAGEN) was used for sequencing reads quality analysis, reads trimming, and de novo assembling. The locations of the protein-coding genes, ribosomal RNAs (rRNAs), and transfer RNAs (tRNAs) were predicted by using MITOS Web Server (Bernt et al. 2013) and identified by alignment with other mitogenome sequence of *Meretrix* shellfish. The AT and GC skew was calculated according to the following formulas: AT skew = (A-T)/(A + T) and GC skew = (G-C)/(G + C) (Perna and Kocher 1995).

The complete mitogenome of *M. lusoria* from Kumamoto is 20,180 bp in length (GenBank Accession No.MT418596), including 13 protein-coding genes, two rRNA genes, and 22 tRNA genes. The nucleotide composition of the mitogenome was 25.73% for A, 42.41% for T, 9.35% for C, and 22.49% for G. The AT content was 68.15% and similar to congeneric species. The AT and GC skewness of mitogenome sequence were −0.245 and 0.412, showing the T-skew and G-skew. The skew statistics (AT and GC) of protein-coding gene were −0.341 and 0.392, showing the same T-skew and G-skew. The mitogenome sequence identity of 13 protein-coding genes is 79.17–88.59% with other *Meretrix* clams. We reconstructed the phylogenetic relationships of Veneroida and relative species based on 12 protein-coding genes (excluding the atp8 gene) of 25 mitogenomes with Maximum-Likelihood (ML) criteria. Bootstrap values (1000 replications) greater than 70% are shown at the branch nodes (Figure 1). The majority of nodes had support values higher than 70% and 17 were up to 100% supported. The *Meretrix* clams were grouped in the same clade and the clade of Veneroida was highly supported. The phylogenetic position of *M. lusoria* (MT418596) is sister to *M. meretrix* (GQ463598), *M. petechialis* (EU145977), and *M. lusoria* (GQ903339). Hsiao et al. (2019) observed that the *cox1* gene sequence of GQ903339 was grouped with the *Meretrix* sp. nov., a new species in Taiwan and southern China. Our results shall provide a better understanding in the evolutionary histories of the Veneroida and relative species. In this study, mitogenomic sequence data will provide useful information for further studies for the population genetics, speciation, biogeography, and conservation of *M. lusoria* in the future.

**Figure 1.**
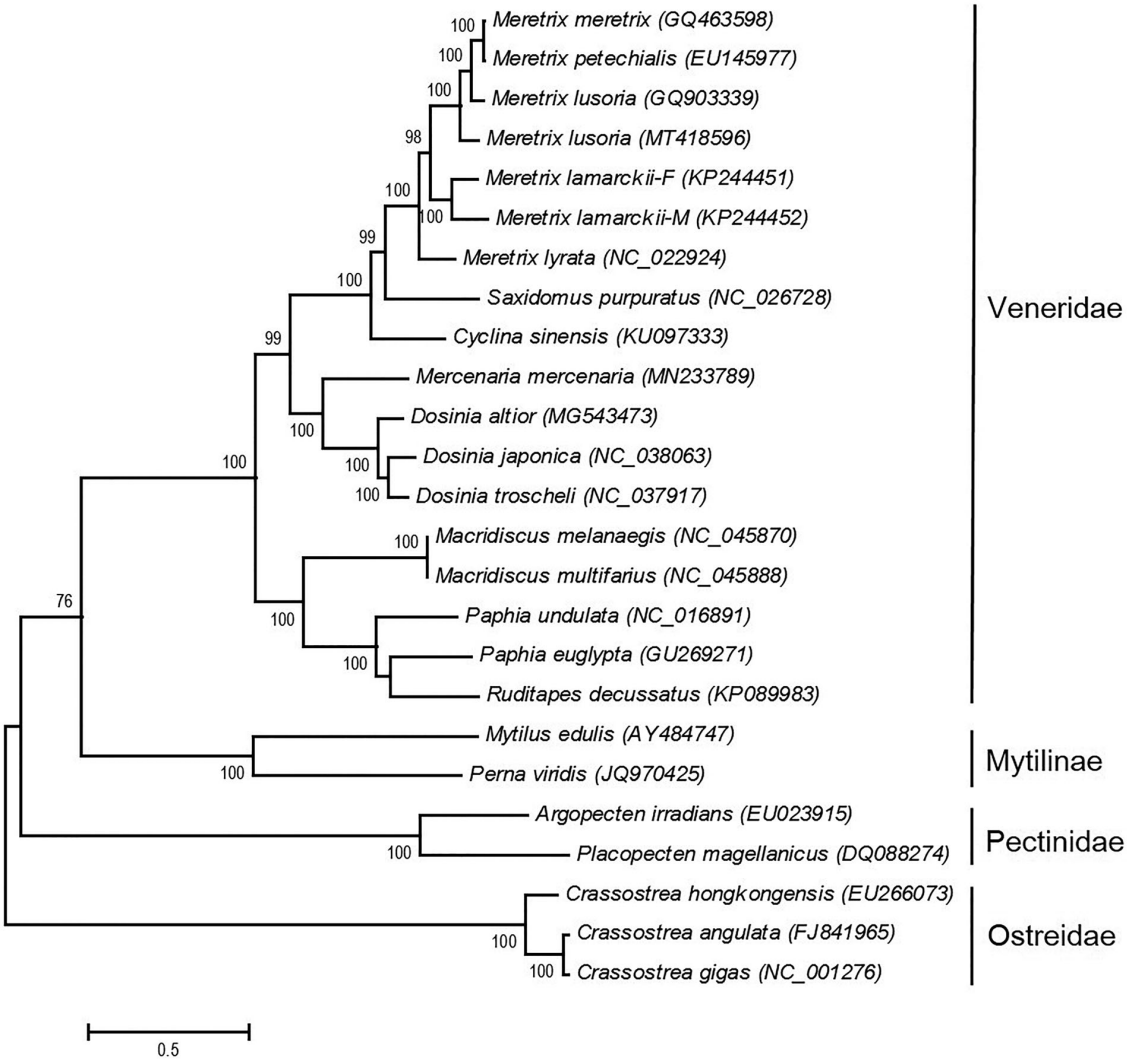
Phylogenetic tree of the 25 Bivalvia species based on the sequence of 12 protein-coding genes. The tree was reconstructed with the Maximum-Likelihood (ML) criteria using MEGA v.6 (Tamura et al. 2013). Bootstrap values (1000 replications) greater than 70% are shown at the branch nodes.

## Data Availability

The data that support the findings of this study are openly available in nucleotide database of NCBI (National Center for Biotechnology Information) at https://www.ncbi.nlm.nih.gov, accession number MT418596.
